# Efficacy and pharmacokinetics of betaine in CBS and cblC deficiencies: a cross-over randomized controlled trial

**DOI:** 10.1186/s13023-022-02567-4

**Published:** 2022-11-14

**Authors:** Apolline Imbard, Artemis Toumazi, Sophie Magréault, Nuria Garcia-Segarra, Dimitri Schlemmer, Florentia Kaguelidou, Isabelle Perronneau, Jérémie Haignere, Hélène Ogier de Baulny, Alice Kuster, François Feillet, Corinne Alberti, Sophie Guilmin-Crépon, Jean-François Benoist, Manuel Schiff

**Affiliations:** 1 Laboratoire de Biochimie-Hormonologie, Hôpital Robert Debré, APHP, Filière G2M, Paris, France; 2 Laboratoire de Biochimie Métabolique, Hôpital Necker-Enfants-Malades, APHP, 149 Rue de Sèvres, Filière G2M, Paris, France; 3 LYPSIS2, Faculté de Pharmacie de Chatenay-Malabry, Université Paris-Saclay, Paris, France; 4 Unité d’Epidémiologie Clinique, Unité de Recherche Clinique, Hôpital Robert Debré, Paris, France; 5 Inserm CIC-EC 1426, Paris, France; 6 UF de Pharmacologie | Service de Microbiologie, Hôpital Jean Verdier, Paris, France; 7 UMR 1137, Inserm, Paris, France; 8 Centre de Référence Maladies Héréditaires du Métabolisme, Hôpital Robert Debré, APHP, Filière G2M, Paris, France; 9 Center for Molecular Diseases, Lausanne University Hospital, Lausanne, Switzerland; 10 Centre d’Investigations Cliniques, Hôpital Robert Debré, APHP, Paris, France; 11 Centre de Compétence Maladies Héréditaires du Métabolisme, Centre Hospitalo-Universitaire de Nantes, Filière G2M, Nantes, France; 12 Centre de Référence des Maladies Métaboliques, Inserm U1256 NGERE, Centre Hospitalo-Universitaire Brabois Enfants, Filière G2M, Vandoeuvre-Les-Nancy, France; 13 Centre de Référence Maladies Héréditaires du Métabolisme, Hôpital Necker-Enfants-Malades, APHP, Filière G2M, Paris, France; 14 Inserm UMR_S1163, Institut Imagine, Université Paris Cité, Paris, France

**Keywords:** Betaine, Homocysteine, Hyperhomocysteinemia, Cystathionine-beta-synthase deficiency, cblC deficiency, Methionine, S-adenosylmethionine, Pharmacokinetics

## Abstract

**Background:**

Betaine is an “alternate” methyl donor for homocysteine remethylation catalyzed by betaine homocysteine methyltransferase (BHMT), an enzyme mainly expressed in the liver and kidney. Betaine has been used for more than 30 years in pyridoxine non-responsive cystathionine beta-synthase (pnrCBS) and cobalamin C (cblC) deficiencies to lower the hyperhomocysteinemia, although little is known about the optimal therapeutic dosage and its pharmacokinetic in these patients.

**Aims:**

We compared 2 betaine doses (100 mg/kg/day vs. 250 mg/kg/day) in children affected by pnrCBS or cblC deficiencies. We also measured the pharmacokinetics parameters after a single dose of betaine (100 or 250 mg/kg) in these patients.

**Methods:**

We conducted a prospective, randomized, crossover clinical trial with blinded evaluation. The primary outcome was the equivalence of total plasma homocysteine (tHcy) concentrations upon one-month oral treatment with betaine at 100 versus 250 mg/kg/day.

**Results:**

Eleven patients completed the study (5 pnrCBS and 6 cblC). tHcy concentrations were equivalent after a one-month treatment period for the two betaine dosages. Multivariate analysis showed a significant effect of betaine dose on methionine (Met) (*p* = 0.01) and S-adenosylmethionine (SAM) concentrations (*p* = 0.006).

**Conclusions:**

Our analysis shows that there is no overt benefit to increasing betaine dosage higher than 100 mg/kg/day to lower tHcy concentrations in pnrCBS and cblC deficiencies. However, increasing betaine up to 250 mg/kg/d could benefit cblC patients through the increase of methionine and SAM concentrations, as low Met and SAM concentrations are involved in the pathophysiology of this disease. In contrast, in pnrCBS deficiency, betaine doses higher than 100 mg/kg/day could be harmful to these patients with pre-existing hypermethioninemia.

*Trial registration*: Clinical Trials, NCT02404337. Registered 23 May 2015—prospectively registered, https://clinicaltrials.gov.

**Supplementary Information:**

The online version contains supplementary material available at 10.1186/s13023-022-02567-4.

## Introduction

**Fig. 1:**
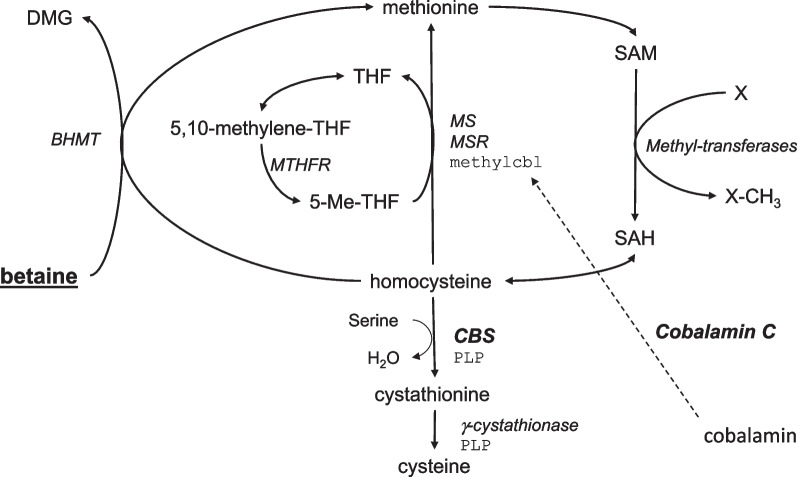
Schematic representation of homocysteine metabolism. 5, 10-methylene-THF: 5,10-methylene-tetrahydrofolate; 5-Me-THF: 5-methyl-tetrahydrofolate; BHMT: betaine homocysteine methyl transferase; CBS: cystathionine beta synthase; MTHFR: methylene-tetrahydrofolate reductase; methylcblc: methylcobalamin; MS: methionine synthase; MSR: methionine synthase reductase; PLP: pyridoxal phosphate; THF: tetrahydrofolate; SAM: S-adenosylmethionine; SAH:S-adenosylhomocysteine

Betaine is a methyl-derived amino acid compound (N, N, N-trimethylglycine) that acts either as an osmolyte to protect cells under stress or as a methyl donor in the remethylation pathway. It is an important nutrient contained in a variety of foods and can also be synthesized in the liver and the kidney mitochondria from its precursor choline through a two-step pathway [1, 2]. In the kidney, betaine is filtrated by the glomerulus and mainly reabsorbed by the proximal tubule. However, the majority of betaine elimination results from its utilization as a methyl donor by the betaine-homocysteine methyltransferase (BHMT) for the remethylation of homocysteine (Hcy) to methionine (Met). This reaction is often considered an “alternate” pathway since BHMT is mainly expressed in the liver and kidney, contrasting with the ubiquitous methionine synthase that catalyzes the same remethylation reaction using methyltetrahydrofolate as a methyl donor (Fig. 1).

Hcy is a toxic compound for endothelium, and hyperhomocysteinemia promotes thrombotic risk, however the underlying mechanisms are complex and still poorly characterized. These mechanisms include oxidative stress, interference with nitric oxide production, and the production of homocysteinylated proteins and DNA. Moreover, S-adenosyl-homocysteine, a precursor of Hcy that accumulates in the setting of hyperhomocysteinemia, acts as a potent inhibitor of methylation reactions (3, 4). The central nervous system can also be affected by hyperhomocysteinemia via endothelial or neuronal toxicity [5, 6]. Therefore, betaine has been used for several decades to lower Hcy concentrations in the treatment of hyperhomocysteinemia due to transsulfuration or remethylation defects.

In cystathionine beta-synthase (CBS) deficiency (OMIM 236,200) [7], betaine is recommended as an additional treatment when low-protein diet and/or vitamin B6 (pyridoxine) supplementation do not achieve to lower Hcy below therapeutic recommendations [8]. Patients with CBS deficiency show wide variability in disease severity and age. The severe pyridoxine non-responsive form typically occurs in childhood and causes cognitive impairment, lens dislocation, marfanoid features, osteoporosis, and thromboembolism [9]. Betaine therapy has significantly reduced homocysteine concentrations and prevented vascular complications in this disorder [10]. Betaine may also act as a chemical chaperone and correct partial misfolding of *CBS* mutants [11–13].

Betaine has also been used for several decades in remethylation disorders (e.g., intracellular cobalamin metabolism and MTHFR deficiencies) [14] and is now recommended in addition to vitamin supplementation [15]. In these disorders, betaine lowers Hcy concentration and contributes to increased Met concentration. Remethylation disorders’ clinical spectrum is broad. These disorders affect central and peripheral nervous systems, leading to developmental and neurocognitive impairments, as well as neuropsychiatric and thrombotic features that are inconsistently associated with hematological abnormalities. Microangiopathy has also been reported mostly in cblC patients [15]. Both hyperhomocysteinemia and methionine depletion leading to decreased remethylation capacities are involved in the pathophysiology of remethylation disorders pleading for betaine use [15].

Despite its use for several decades, little is known about the optimal therapeutic doses and pharmacokinetics of betaine in transtranssulfuration and remethylation defects. The recommended dose of betaine in the pediatric population begins at 100 mg/kg/day. However, there are conflicting recommendations in the literature regarding the maximum amount, typically up to 250 mg/kg/day and sometimes beyond but without a clear demonstration of the benefits of dose escalation [16]. Most of the pharmacokinetics data were obtained in healthy adult volunteers [17, 18] and not in patients with homocystinurias.

The present study aimed at assessing the relative efficacy of one-month treatment with two doses of betaine (100 mg/kg/day *vs* 250 mg/kg/day) in children affected with pyridoxine non-responsive CBS (pnrCBS) deficiency or cblC defect based on the effect on plasma total homocysteine (tHcy) concentrations. A secondary aim was also to determine the pharmacokinetic parameters of betaine in these patients.

## Materials and methods

### Study design

This prospective, randomized, equivalence, two-period, and cross-over clinical trial open with blinded evaluation was conducted at three pediatric departments in France. Each of the two one-month periods consisted of oral treatment with betaine at 100 mg/kg/day or 250 mg/kg/day with a one-week wash-out period between the treatment periods. The completion and reporting of the trial have complied with CONSORT 2010 guidelines.

### Study oversight

The protocol was approved by national ethics committees (Comité de Protection des Personnes [CPP IDF III], the French National Drug Safety Agency [ANSM, EudraCT 2014-003643-36] and the Franch data protection authority [Comission Nationale de l’Informatique et des Libertés, CNIL]). Funding was provided by the French Health ministry (AOR13016). The trial was registered at ClinicalTrials.gov (NCT02404337).

### Participants

Patients were eligible for inclusion if aged 1–18 years and diagnosed with either pyridoxine non-responsive CBS (pnrCBS), or cblC deficiencies confirmed enzymatically and/or molecularly, treated continuously for at least one year. Exclusion criteria were pyridoxine-responsive CBS deficient patients and pregnant females.

### Intervention

Subjects were recruited in each center, included in the trial, and then investigated in the same center (Clinical Investigation Center (INSERM CIC1426) at the Robert Debré Hospital, Paris, France). Each patient was randomly assigned to one group. The first trial period was followed by a one-week wash-out period to mitigate the possibility of carry-over effects and reduce the likelihood that treatment from the prior first period influenced outcome measures of the current second period.

From day three until the end of the month, they received betaine at 100 mg/kg/day or 250 mg/kg/day as a twice-daily dose. Fasting blood samples were collected before morning betaine administration at the end of the one-month treatment for each dose. All the patient's usual treatment, including protein restriction (apart from betaine), remained unchanged throughout the protocol.

### Outcomes

The primary outcome was plasma concentrations of total homocysteine at one month upon oral treatment with betaine at 100 mg/kg/day compared with 250 mg/kg/day in the same individual.

Secondary outcomes included plasma concentrations of betaine, dimethylglycine, sarcosine, methionine, S-adenosylmethionine (SAM), S-adenosylhomocysteine (SAH), creatine, guanidinoacetate, choline, ethanolamine, methylmalonic acid (MMA) and urinary concentrations of betaine, dimethylglycine, sarcosine, homocysteine, methionine, creatine, guanidinoacetate, and MMA before and after one month of treatment. The known side effects of betaine were also recorded in clinical outcomes, notably gastrointestinal disorders. All laboratory parameters were centralized and analyzed at the Biochemistry laboratory (Robert Debré Hospital, Paris, France).

### Metabolites measurement

Blood containing EDTA as an anticoagulant was drawn at different time points and immediately centrifuged. Plasma was subsequently collected and frozen at − 80 °C until analysis. SAM, SAH, choline, d_9_-choline, betaine, d_9_-betaine, dimethylglycine (DMG), ethanolamine (EA), sarcosine, d_3_-sarcosine, methionine, L-homocystine, dithiothreitol (DTT), creatine, d_3_-creatine, guanidinoacetate (GAA), ^13^C_2_-GAA and MMA were obtained from Sigma-Aldrich (Saint Louis, USA). d_3_-SAM, d_6_-dimethylglycine, d_4_-ethanolamine, d_3_-methionine, and d_8_-DL-homocystine were purchased from CDN-isotope (Pointe-Claire, Canada). D_4_-SAHwas bought by cayman chemical (Ann Arbor, USA).

Free choline, ethanolamine, creatine, GAA, and MMA were assayed after deproteinization by an LC–MS/MS method adapted from Holm et al. [19], Imbard et al. [20], and Cognat et al. [21]. The analytical system consisted of an Acuity UPLC I Class system (Waters, Milford, USA) coupled with a Xevo-TQD (Waters, Milford, USA) with an Atlantis HILIC analytical column (2.1 × 100 mm, 3 µm) (Waters, Milford, USA).

SAM, SAH, tHcy, Met, betaine, DMG, and sarcosine were measured using new LC–MS/MS developed in our laboratory (detailed in the Additional file 1).

### Sample size

Sample size calculation was conditional to the number of potentially eligible subjects per year in the three centers for these rare diseases, estimated as 18 subjects.

### Randomisation

The treatment sequence (100 then 250 vs. 250 then 100) was randomly allocated via a randomization list, previously established by the Clinical Epidemiology Unit of the Robert Debré Hospital, Paris, France, using random blocks of variable size. Stratification on the type of pathology was applied to limit bias. All biological samples were centralized and frozen, and the measurements taken at the end of the study were blinded from the allocated arm.

### Statistical methods

Descriptive analysis was used to characterize subjects at baseline (all subjects), then evolution and treatment tolerance during the study (considering two groups among betaine dosage 100 mg/kg/day or 250 mg/kg/day) and by disease type (CBS or cblC deficiency). Data was expressed as medians with [min; max] for continuous variables and numbers with percentages for categorical variables.

Mean differences were calculated and tested to verify the absence of a carryover effect between the two sequences (100 mg/kg/day then 250 mg/kg/day vs. 250 mg/kg/day then 100 mg/kg/day) periods. In the case of significant interaction indicating the presence of carryover, only results from the first period were analyzed.

The ratio of plasma tHcy concentration at 30 days in the 100 mg/kg/day and 250 mg/kg/day groups was calculated to test the primary equivalence outcome. As a sensitivity analysis, a two one-sided tests (*TOST*) approach was also used to test equivalence [22]. Equivalence was declared if the 90% confidence interval of this ratio was between 0.8 and 1.25, taking into account the logarithm of the dosage.

The evaluation in plasma tHcy measured at one month by oral treatment with betaine between 100 and 250 mg/kg/day was tested using a 2-sided Type 3 *F*-test of the treatment effect in a linear mixed-effect regression model for repeated measures. The model included fixed effects for treatment dosage (100 or 250), period (1 or 2), the interaction between treatment dosage and period (dosage*period), and the disease type (CBS or cblC deficiency) with random effects for the subject.

Similar linear mixed-effect regression models for repeated measures were used to evaluate the secondary metabolic outcomes.

Analyses were performed on the intention-to-treat basis, with a *p*-value < 0.05 considered significant. Data management and statistical analysis were performed using SAS software, Institute Inc., Cary, NC, version 9.4.

### Pharmacokinetic analysis

At the beginning of each one-month period each patient received the daily dosage (100 mg/kg/day or 250 mg/kg/day) in a single administration to evaluate the pharmacokinetics of betaine over 48 h. Samples were collected before betaine administration (H0) and 2 h, 6 h, 12 h, 24 h, and 48 h after the first single-dose betaine administration. Plasma concentrations of betaine, dimethylglycine, sarcosine, and urinary concentrations of betaine, dimethylglycine, and sarcosine were measured.

According to standard procedures, individual concentration–time data of betaine were evaluated using a non-compartmental (NCA) approach (PKsolver, version 2.0, China Pharmaceutical University, Nanjing, China).

The total area under the plasma concentration *vs.* time curves from zero to 48 h (AUCP, 0–48 h) were calculated using the linear trapezoidal rule. The rate constant kel and corresponding half-lives (t1/2) were estimated by a least-squares fit of data points (log concentration–time) in the terminal phase of the decline. The maximum concentration after the first dose (Cmax) and the time at which it appears (Tmax) were obtained from the observed data. The Cmax and AUC ratios after a 250 mg/kg and 100 mg/kg dose were calculated for each patient.

Apparent total body clearance of betaine (CL/F) was calculated as the ratio between the dose of betaine (Dose) and the corresponding AUCP, 0–48 h, with F, the bioavailability of the molecule. The apparent betaine steady-state volume of distribution (VSS /F) was obtained from (Dose. MRT)/(AUCP, 0–48 h), where MRT is the mean residence time of the molecule (h).

## Results

### Patients

**Fig. 2:**
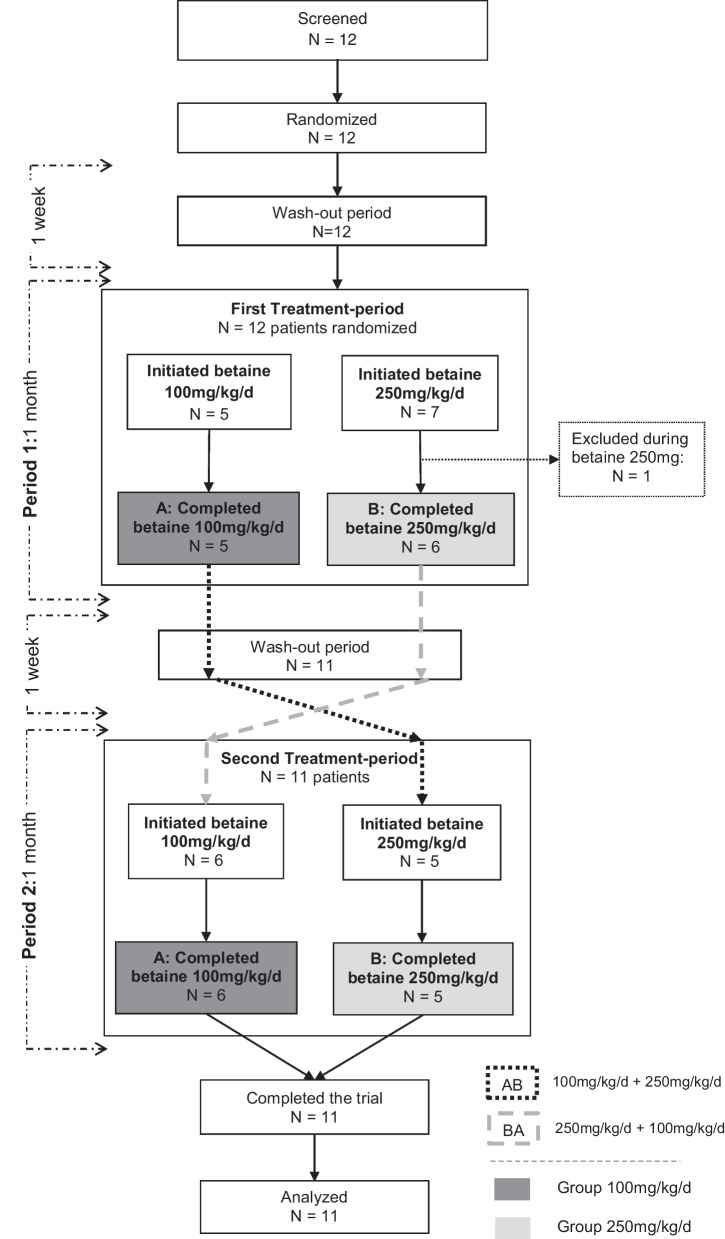
Flow-chart of the cross-over study design

Twelve patients were recruited for the study (pnrCBS N = 5; cblC N = 7) between July 2015 and October 2017 (Fig. 2). One patient (cblC) withdrew his consent during the first period of the trial prior to any blood draws. Eleven subjects completed the study (pnrCBS N = 5; cblC N = 6) for efficacy, pharmacokinetics, and tolerance.

Baseline characteristics of patients are presented in Table 1 and Additional file 1: Tables S1, S2 and S3. The median age at diagnosis was 2.3 months, with two cblC patients diagnosed in the neonatal period (before 25 days of life) and all pnrCBS patients diagnosed after 25 days of life. For all patients, the diagnosis was suspected based on suggestive clinical signs and laboratory parameters (Met, Hcy) and confirmed either by molecular or enzymatic studies (Additional file 1: Table S1). At inclusion, the median age was 9.4 [1.5–17.8] years. All patients (N = 11) were treated with betaine at protocol inclusion with various dosages (Table 1). PnrCBS patients were all under low-protein diet (14 [11–17] g of natural protein per day + 31.2 [15–50] amino acid mixture per day) and *per oral* vitamin B12 supplementation: 3/5 patients cyanocobalamin or 2/5 hydroxocobalamin, 4/5 were supplemented with pyridoxine, 4/5 with cysteine and 3/5 with folinic acid (Additional file 1: Table S2). cblC patients were all treated with intramuscular ± *per oral* hydroxocobalamin, 4/6 patients were treated with methionine supplementation, 5/6 with folinic acid, and 3/6 with pyridoxine (Additional file 1: Table S2). The main clinical features of the patients are available in Additional file 1: Table S3.

**Table 1:** Population description at baseline

Patient number	Disease	Sex	Age at diagnosis	Age at study (years)	Betaine (mg/kg/days) at inclusion
1	pnrCBS	M	6.8 y	14.41	**76.3**
2	pnrCBS	F	2.1 y	9.62	**107.5**
3	cblC	F	3 w	3.62	**137.9**
4	cblC	M	18 d	4.22	**88.9**
5	cblC	F	3 w	9.35	**190.7**
6	cblC	M	1 m	17.8	**150.9**
7	cblC	M	2 m	6.55	**272.1**
8	pnrCBS	M	4.1 y	13.4	**92.6**
9	pnrCBS	M	3.2 y	6.78	**160.0**
10	pnrCBS	F	6.8 y	13.25	**80.5**
11	cblC	M	1 m	1.46	**94.3**

M: Male; F: female; d: days; w: weeks; m: months; y: years

### Efficacy

#### Primary endpoint: plasma total homocysteine (tHcy) after one month of treatment

Carryover effects on plasma tHcy were analysed: order and period effects and interaction dose and period, did not show any carry-over effect (p = 0.28) (Additional file 1: Fig. 1).

**Fig. 3:**
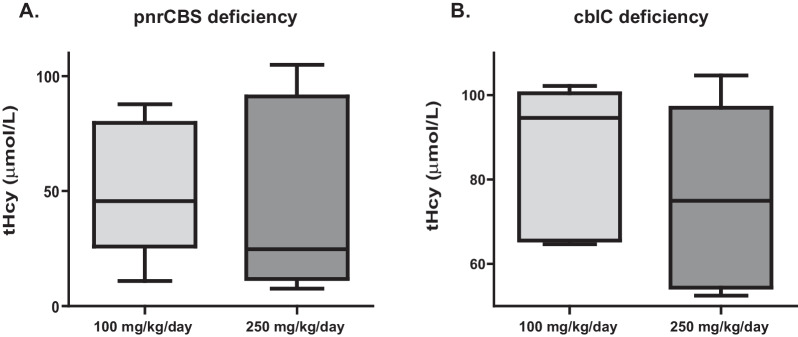
Plasma total homocysteine (tHcy) after 1 month of treatment at 100 mg/kg/day (light grey) or 250 mg/kg/day (dark grey) of betaine in pyridoxine non responsive CBS (pnrCBS) (**A**) and cblC patients (**B**). No significant differences were observed with betaine dose (− 9.79 [− 23.99; 4.41], *p* 0.14) or pathology (19.97 [− 15.76; 55.71], *p* 0.23) on plasma tHcy concentrations after one month of treatment

Plasma tHcy concentrations in cblC and pnrCBS patients with both betaine doses are presented in Table 2, Fig. 3 and Additional file 1: Fig. S2. The mixed model did not show any effect of betaine dose (− 9.79 [− 23.99; 4.41], *p* = 0.14) or disease type (pnrCBS or cblC) (19.97 [− 15.76; 55.71], *p* = 0.23) on plasma tHcy concentrations after one month of treatment. TOST test confirmed these results with an IC_90_ of tHcy ratio between the two dosages at one month at [1.01; 1.14], *p* = 0.0003.

**Table 2:** Metabolites concentrations of other 1-C metabolism (secondary endpoints) after 1 month of betaine treatment (100 mg/kg/day vs. 250 mg/kg/day)

	CBS deficiency		cblC deficiency	
	N = 5		N = 6	
	100 mg/kg/d	250 mg/kg/d	100 mg/kg/d	250 mg/kg/d
Plasma tHcy (µmol/L)	45.7 [11.0; 87.6]	24.7 [7.6; 104.8]	94.6 [64.7; 102.2]	75.0 [52.5; 104.7]
Plasma Met (µmol/L)	57.1 [38.3; 68.9]	77.7 [59.7; 269.0]	17.6 [9.7; 25.4]	27.6 [15.7; 32.4]
Plasma SAM (nmol/L)	145.3 [106.3; 205.4]	285.5 [217.2; 456.5]	163.5 [58.6; 235.0]	179.2 [109.9; 369.2]
Plasma SAH (nmol/L)	38.6 [26.2; 74.3]	44.2 [34.7; 287.2]	32.4 [20.5; 39.5]	32.6 [21.8; 56.3]
Plasma SAM/SAH (nmol/L)	3.4 [2.5; 4.1]	5.0 [1.6; 8.2]	5.3 [2.9; 6.0]	5.8 [3.2; 6.9]
Plasma choline (µmol/L)	9.6 [7.7; 14.1]0	11.3 [6.4; 12.9]	11.5 [8.9; 15.8]	10.6 [8.7; 18.5]
Plasma ethanolamine (µmol/L)	10.0 [5.2; 15.5]	8.2 [5.8; 12.1]	13.2 [6.0; 17.8]	10.9 [5.1; 21.6]
Plasma choline/ethanolamine	1.1 [0.6; 1.9]	1.4 [0.6; 2.0]	0.9 [0.5; 2.6]	0.8 [0.5; 3.6]
Plasma creatine (µmol/L)	22.8 [18.7; 34.2]	20.8 [16.5; 38.6]	62.6 [21.2; 96.2]	27.0 [14.5; 65.6]
Plasma GAA (µmol/l)	2.1 [1.0; 2.6]	2.2 [1.3; 2.5]	1.5 [1.3; 2.2]	1.4 [1.1; 2.2]
Plasma creatine/GAA	12.8 [7.3; 33.9]	13.9 [6.7; 19.7]	37.7 [ 9.6; 65.1]	18.2 [6.5; 50.3]
Plasma MMA (µmol/L)			45.8 [16.2; 105.1]	39.9 [12.4; 107.0]
Plasma Betaine (µmol/L)	137.5 [62.4; 260.1]	405.4 [224.1; 635.4]	264.9 [36.7; 983.3]	941.9 [158.8; 1559.3]
Plasma dimethylglycine (µmol/L)	42.3 [29.0; 266.5]	247.1 [123.3; 398.9]	54.1 [7.8; 202.8]	81.9 [42.2; 226.1]
Plasma sarcosine (µmol/L)	22.0 [19.0; 27.6]	39.1 [34.2; 46.6]	12.0 [2.8; 16.5]	18.3 [8.9; 28.7]
Urine tHcy (µmol/mmol creatinine)	7.8 [1.2; 9.7]	3.9 [0.5; 13.8]	20.3 [13.8; 36.2]	21.5 [11.6; 30.0]
Urine Met (µmol/mmol creatinine)	3.0 [1.9; 3.6]	4.4 [1.8; 13.1]	2.9 [0.8; 4.0]	4.0 [3.1; 5.9]
Urine creatine (µmol/mmol creatinine)	19.0 [10.0; 69.0]	17.0 [14.0; 65.0]	299.5 [14.0; 599.0]	24.0 [15.0; 705.0]
Urine GAA (µmol/mmol creatinine)	36.0 [21.0; 64.0]	39.0 [30.0; 42.0]	50.0 [34.0; 109.0]	28.5 [15.0; 96.0]
Urine creatine/GAA	0.7 [0.2; 1.6]	0.4 [0.4; 1.7]	4.0 [0.4; 8.9]	0.9 [0.5; 7.3]
Urine MMA (µmol/mmol creatinine)			287.0 [113.7; 1346.0]	307.5 [142.5; 1174.0]

Data are presented as mediane [min; max]

#### Secondary endpoints: intermediates of homocysteine metabolism after one month of treatment

Plasma and urinary concentrations of all metabolites of homocysteine metabolism after one month of betaine treatment at 100 mg/kg/day and 250 mg/kg/day are presented in Table 2.

**Fig. 4:**
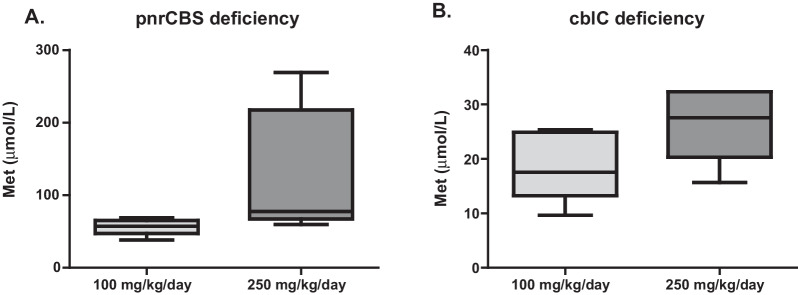
Plasma methionine (Met) after 1 month of treatment at 100 mg/kg/day (light grey) or 250 mg/kg/day (dark grey) of betaine in pyridoxine non responsive CBS (pnrCBS) (**A**) and cblC patients (**B**). Multivariate analysis show a significant effect of interaction between betaine dose × period (0.73 [0.09; 1.37], *p* 0.03) and type of disease (CBS vs. cblC deficiency, − 1.40 [− 2.61; − 0.19], *p* 0.03)

Multivariate analysis showed a significant effect of interaction between betaine dose × period (0.73 [0.09; 1.37], *p* = 0.03) and type of disease (CBS *vs* cblC deficiency, − 1.40 [− 2.61; − 0.19], *p* = 0.03) on Met concentrations (Fig. 4). In the multivariate analysis, SAM concentrations were also significantly influenced by betaine dose (*p* = 0.006) (Additional file 1: Fig. S3). The mixed model did not show any effect of betaine dose or pathology on SAH and the SAM/SAH ratio after one month of treatment (Additional file 1: Fig. S3).

### Tolerance

Eight patients (4 pnrCBS/4 cblC patients) presented one to 5 minor adverse events: 3 during the treatment with 100 mg/kg/day, 3 during the treatment with 250 mg/kg/day, and 2 with both dosages. One patient reported minor adverse events that were already present before the start of the study. This patient also had seizures during the wash-out period.

Minor adverse events reported with 100 mg/kg/day of betaine were gastrointestinal disorder (N = 3: diarrhea, vomiting, abdominal pain), fever (N = 2), headache (N = 1), muscular pain (N = 1), and intercurrent infectious disease (N = 1 varicella).

Minor adverse events reported with 250 mg/kg/day of betaine were gastrointestinal disorder (N = 3: diarrhea, vomiting, abdominal pain), intercurrent infectious disease (N = 2 ringworm, angina), and fever (N = 2).

### Pharmacokinetics parameters

**Fig. 5:**
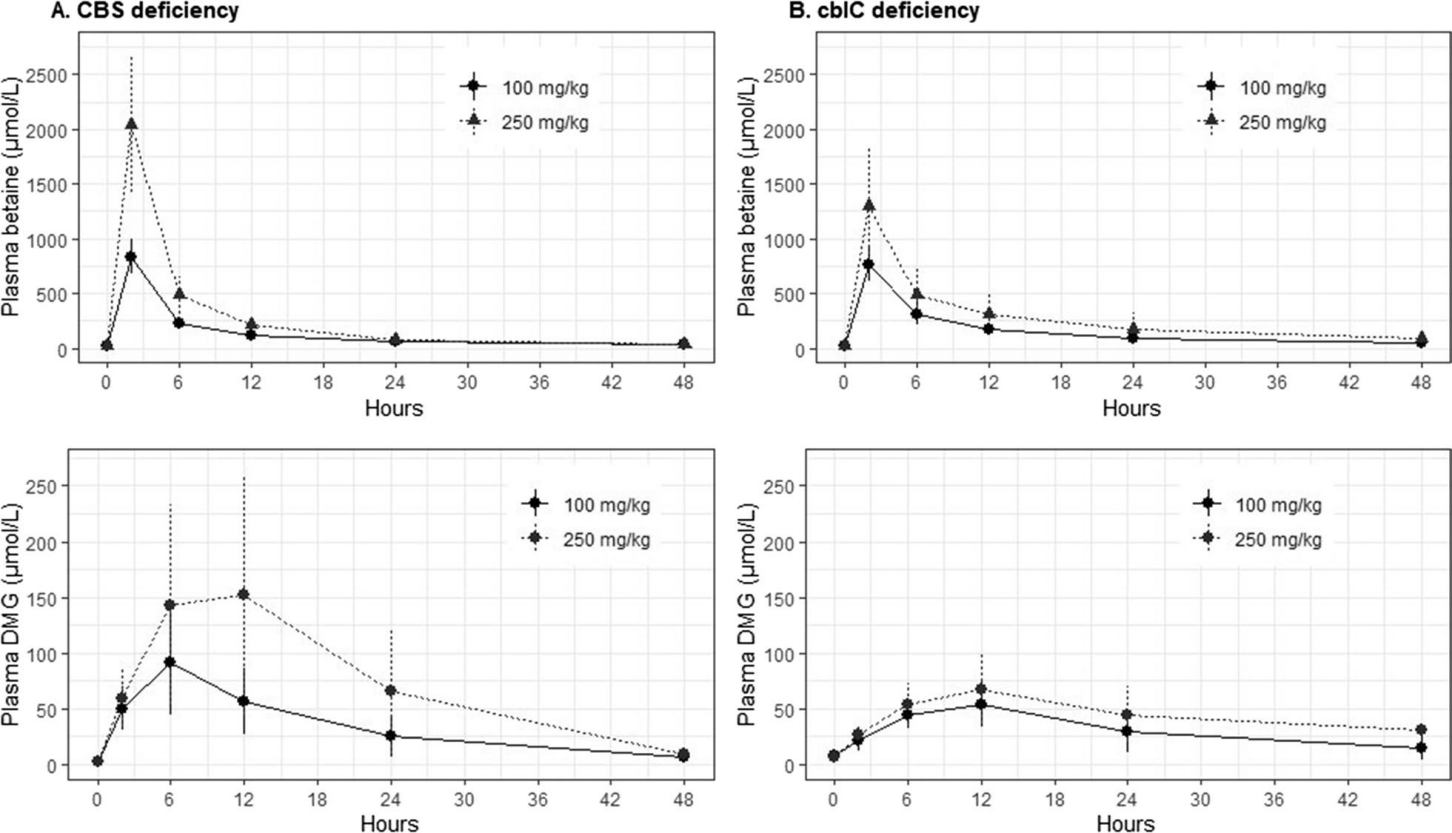
Pharmacokinetic parameter of betaine and dimethylglycine after oral betaine dose of 100 mg/kg or 250 mg/kg in CBS and cblC deficit (mean ± SD)

Pharmacokinetic parameters of betaine and dimethylglycine are reported in Table 3. The AUC and Cmax of betaine were proportional between the two tested doses (expected ratio of 2.5) in pnrCBS patients (Fig. 5A) but not for cblC patients (Fig. 5B). CL/F and V/F of betaine in patients with pnrCBS were similar regardless of dosage, whereas these parameters appeared to increase in patients with cblC. The AUC of DMG was comparable between the 2 conditions, but DMG Cmax was lower and DMG half-life was much longer in cblC patients.

**Table 3:** Detailed pharmacokinetic parameters

	CBS	cblC	CBS	cblC	CBS	cblC
	100 mg/kg	100 mg/kg	250 mg/kg	250 mg/kg	Ratio 250/100	Ratio 250/100
	N = 5	N = 5	N = 5	N = 6	N = 6	N = 6
AUC_P,0-48 h,betaine_ (µmol h/L)	5522 [4909; 9317]	7485 [4843; 11907]	13,276 [6454; 14907]	10,389 [7826; 29243]	2.16 [1.30; 2.70]	1.32 [1.22; 2.46]
C_max,betaine_ (µmol/L)	849 [681; 1048]	717 [585; 1000]	2074 [1001; 2563]	1136 [827; 2356]	2.80 [1.47; 3.00]	1.61 [1.17; 2.36]
T_max,betaine_ (h)	2	2	2	2		
CL_betaine_/F (L/h kg^−1^)	0.15 [0.09; 0.17]	0.11 [0.07; 0.18]	0.16 [0.14; 0.33]	0.21 [0.07; 0.27]		
V_ss,betane_/F (L kg^−1^)	1.45 [1.2; 1.98]	1.56 [1.04; 1.86]	1.29 [1.15; 3.41]	2.34 [1.15; 3.49]		
t_1/2, betaine_	16.7 [13.8; 20.3]	18.3 [10.9; 24.2]	12.3 [10.0; 16.3]	18.4 [9.43; 25.9]		
AUC_P, 0–48 h, DMG_ (µmol h/L)	1627 [1149; 2496]	1408 [669; 2247]	2269 [1380; 7055]	2022 [1120; 2470]	1.51 [1.12; 2.99]	1.37 [1.08; 1.67]
C_max,DMG_ (µmol/L)	77.4 [53.6; 169]	58.2 [31.9; 73.3]	106 [76.8; 288]	69.8 [42.1; 125.7]	1.43 [1.00; 4.12]	1.32 [1.08; 1.71]
T_max,DMG_ (h)	6 [6; 24]	12 [6; 12]	12 [6; 12]	12 [6; 48]		
t_1/2,DMG_	10.5 [8.82; 13.5]	19.5 [13.9; 25.1]	8.64 [7.39; 12.5]	16.0 [10.6; 25.5]		

Data are presented as mediane [min; max]. AUC_P,0–48 h_: Total area under the plasma concentration *vs* time curves from zero to 48 h; C_max_: Maximal concentration; T_max_: Time at maximal concentrations; CL/F CL: Apparent total body clearance; V_SS:_ Apparent steady-state volume of distribution; t1/2: Half-life

## Discussion

This study provides data on the relative efficacy of two different dosages of betaine on plasma tHcy in pediatric patients affected by pnrCBS and cblC deficiencies. As already suggested [8, 15] but never demonstrated, we established the equivalence between 100 mg/kg/day and 250 mg/kg/day of betaine on plasma tHcy concentrations in pnrCBS and cblC patients. We also found a predictable dose-dependent increase in mean Met and SAM plasma concentrations after one month of treatment in pnrCBS and cblC patients. We also determined the pharmacokinetics of betaine in cblC patients.

The efficacy of betaine treatment is challenging to evaluate, as betaine is never used alone but in association with various treatments. In pnrCBS, a protein-restricted diet with supplementations of low doses of folate and vitamin B12 if deficient are recommended [8]. Additional therapy with betaine can help patients who have difficulties adhering to dietary restrictions and reaching satisfactory metabolic control. In cblC, the cornerstone of treatment is parenteral hydroxocobalamin associated with betaine and possibly folate and carnitine supplementations [15]. All the treatments (including diet) remained unchanged throughout the study except betaine, and we designed a cross-over study that allowed limiting variations using the patient as its control. No study exists assessing the relative efficacy of betaine using different dosages, possibly because of the risk of thromboembolism related to the possibility of rapid plasma tHcy rise at the discontinuation of betaine treatment. To limit the impact of betaine wash-out on tHcy levels, we limited the wash-out time to a minimal period of 8 days that is theoretically sufficient according to available pharmacokinetics data [17, 18, 23]. We validated that the wash-out period was long enough by the absence of a carry-over effect between the two doses of betaine (Additional file 1: Fig. S1). The two doses (100 and 250 mg/kg/day) of betaine were chosen based on clinical practice and expert opinion data that proposed to start with 100 mg/kg/day (ref Morris guidelines). Regardless, in clinical practice, betaine dosage is often increased up to 250 mg/kg/day and sometimes more though with only anecdotal evidence of biochemical benefit [8, 15, 16, 24].

A critical point of our study design was whether a one-month-period treatment could be sufficient to observe an effect on plasma tHcy concentrations. We assumed that this time period would be appropriate based on data showing a rapid effect of betaine on plasma tHcy concentrations before one month of treatment [7].

In pnrCBS deficiency, our data suggests that increasing betaine to dosage greater than 100 mg/kg/day does not provide further reduction of plasma tHcy concentrations although there might be differences in some patients (Additional file 1: Fig. S2). However, care must be taken because increasing betaine dosage induces a proportional increase in Met concentrations. This increase is not sought and is not desirable for patients who have Met concentrations that are already permanently elevated due to CBS deficiency. Betaine supplementation without sufficient dietary methionine restriction can induce severe hypermethioninemia in CBS patients. Clinical signs of increased intracranial pressure and brain edema can be observed in patients with plasma Met concentrations close to 1000 µmol/L [25].

In contrast with pnrCBS deficiency, increasing Met and SAM concentrations are desirable in cblC deficiency, in which patients exhibit low Met and SAM concentrations inherent to the remethylation defect. These low concentrations are probably involved in the pathophysiology of the disease. Unfortunately, there is no non-lethal cblC deficient mouse model to investigate the involvement of low Met and SAM concentrations in the disease pathophysiology [26, 27]. However, the low Met and SAM concentrations have been associated with hypomyelination in patients with MTHFR deficiency (sharing remethylation dysfunction with cblC defect) [28, 29]; in the Mthfr-/- mouse model, the decrease of SAM concentrations were correlated to the reduction o glutamate and γ-aminobutyric acid in brain tissue [30].

Very few studies have investigated the pharmacokinetics of betaine and its metabolite, DMG. Data is available from healthy adult volunteers, which does not allow a comparison with the patient population [17, 18]. Only one study was performed on six patients aged 6–17 years with CBS deficiency and receiving a single dose of 100 mg/kg betaine [23]. Cmax was not reported, but the apparent total body clearance (CL/F) and elimination half-lives reported were similar to those observed in our study (Table 4). There is no published pharmacokinetic data on betaine in cblC patients but very few in MTHFR deficiency. Schwahn and collaborators reported pharmacokinetics of betaine and DMG in 2 MTHFR patients aged 5.8 and 10.2 years treated with 50 mg/kg betaine [18]. The Cmax (normalized to the dosage) after one dose of betaine was proportionally higher than those obtained herein (Table 4). Both patients' CL/F and distribution volume (Vd/F) were comparable to our results. However, DMG peak concentrations (Cmax) in these two children were proportionately lower than in our patients (11 and 10 µmol/L for a 50 mg/kg betaine dose *vs* a median of 58.2 mg/L for a 100 mg/kg betaine dose, respectively). Our results, in line with the previsously published data, argue in favor of a twice-daily dose of betaine as it is already the case in clinical practice.

**Table 4:** Comparison of pharmacokinetics data of betaine with the litterature

		Age (y)	Dose (mg/kg)	Cmax (µmol/L)	AUC (µmol h/L)	t_1/2,elim_ (h)	CL/F (L/h kg^−1^)	V/F (L/h kg^−1^)
CBS deficiency								
Matthews et al. [23]	n = 6	14.5 [6–17]^a^	100	na	na	11.8 [8.0 – 26.3]^a^	0.174 [0.054 – 0.210]^a^	na
Current study	n = 5	13.3 [6.8–14.4]^a^	100	849 [681–1048]^a^	5522 [4909; 9317]^c^	16.7 [13.8–20.3]^a^	0.15 [0.09–0.17]^a^	1.45 [1.2–1.98]^a^
			250	2074 [1001–2563]^a^	13,276 [6454; 14907]^c^	12.3 [10.0–16.3]^a^	0.16 [0.14–0.33]^a^	1.29 [1.15–3.41]^a^
cblC/MTHFR deficiency								
Schwahn et al. [18]	n = 1	5.8	50	506	1450	6.63	0.297	1.726
	n = 1	10.2	50	556	4230	23.2	0.102	1.907
Current study	n = 6	5.4 [1.5–17.8]^a^	100	717 [585–1000]^a^	7485 [4843; 11907]^a^	18.3 [10.9–24.2]^a^	0.11 [0.07–0.18]^a^	1.56 [1.04–1.86]^a^
			250	1136 [827–2356]^a^	10,389 [7826; 29243]^a^	18.4 [9.43–25.9]^a^	0.21 [0.07–0.27]^a^	2.34 [1.15–3.49]^a^

Cmax: Betaine maximal concentration; AUC: betaine area under the curve; t_1/2,elim_: betaine elimination half-life; CL/F: betaine apparent clearance, V/F: betaine apparent volume of distribution; na: non available

^$^Doses in mg/kg are calculated using the mean weight of the patients

^a^Median [range]

In our study, no samples were drawn between 0 and 2 h, in contrast with the two other studies already mentioned, in which numerous blood samples were taken after the oral intake of betaine [18, 23]. Therefore, we cannot exclude an underestimation of our Cmax and AUC values, especially for betaine parameters that could affect the apparent clearance and volume of distribution values.

We observed that the Cmax and AUC of betaine vary with the dosage in pnrCBS but not in cblC. In addition, in cblC, the apparent clearance (CL/F) and apparent volume of distribution (V/F) of betaine appeared to be higher when a dose of 250 mg/kg versus 100 mg/kg was administered (Table 3). This difference could be explained by a lower bioavailability (F) in cblC patients when the dosage increased. This non-proportional AUC may be related to lower absorption of betaine increased dosages and/or an increase in its elimination. Two types of transporters have been identified for betaine absorption at the apical pole of intestinal epithelium cells: the saturable H^+^ dependant PAT1 transporter (encoded by *SLC36A1* gene) and the Na^+^/Cl^−^ dependant cotransporter SIT1 (gene *SLC6A20*) [31–33]. The absorption of betaine at the basolateral pole of epithelial cells is supposed to be driven by the BGT1 transporter [33]. However, there is no data regarding differences in betaine absorption in pnrCBS or cblC deficiency. One hypothesis to explain a lower absorption of betaine in cblC could be a different modulation of the intestinal transporters of betaine in cblC *vs* pnrCBS. However, there is little data concerning the regulation of betaine transport, such as the regulation of PAT1 activity by low pH [33] or the increased intake of betaine correlated with sodium luminal sodium concentration [34].

In pnrCBS deficiency, the Cmax and AUC were not dose-proportional for DMG (ratio of 1.4 between the two doses instead of 2.5) as they were for betaine. Since no differences were observed in betaine and DMG urinary excretion between pnrCBS and cblC (data not shown), these lower ratios for DMG could suggest a lower or slower catabolization of betaine at the highest dosage through the BHMT in pnrCBS *vs* in cblC. A lower or slower betaine catabolization could be due to a decrease in mRNA and protein expression associated with a lower enzyme activity of BHMT; such decreases has been observed in various mouse models of CBS deficiency compared to wild-type mice [35–37]. This decrease in BHMT expression and activity has been linked to the hypermethioninemia observed in pnrCBS that could increase oxidative stress by reducing cysteine and glutathione production [37].

Moreover, some studies have suggested that betaine concentrations were associated with polymorphisms in genes involved in its metabolism [38, 39]. Large studies are therefore needed to understand if these polymorphisms could impact the efficacy of betaine treatment in order to adapt betaine dose to patient genotype.

Despite this study’s optimized design, it has several limitations, such as the number of patients and their heterogeneity in age. The low number of patients is linked to the rarity of these diseases, and we chose to only include patients with severe forms of these diseases. Specifically, we only included B6 unresponsive CBS deficiency forms that are associated with more severe phenotypes [9] and cblC patients carrying two severe variants leading to a premature stop codon (nonsense variants or frameshift variants) (Additional file 1: Table S1) and severe early-onset phenotype (Additional file 1: Table S3).

Our study was also limited in that it was only based on laboratory parameters outcomes. We hypothesized that clinical outcomes were not relevant since the neurological evolution is slow under treatment and cannot be modified during a one-month period; similarly, thromboembolic events are too rare under treatment to obtain statistical power in such a limited number of patients (no thromboembolic event was observed during the study).

In conclusion, our study demonstrated that increasing betaine dosage at 250 mg/kg/day in pnrCBS and cblC deficient patients had no significant effect on plasma tHcy concentrations after one month of treatment. However, it allowed increasing both Met and SAM plasma concentrations, raising the question of its benefit in cblC deficient patients in whom the decrease of Met or/and SAM plays a pathophysiological role. Further and larger trials should be considered to confirm these findings.

## Take home message

Increasing betaine from 100 to 250 mg/kg/day has no significant effect on plasma total homocysteine but increase methionine and S-adenosylmethionine in CBS- and cblC-deficient patients therefore pleading for pleading for 250 mg/kg/day solely in cblC patients.

## Supplementary Information

**Additional file 1: Table S1**: Patients’ characteristics. **Table S2**: Detail of patient current treatment. **Table S3**: Clinical data at inclusion. **Figure S1**: Carry-over effect. **Figure S2**: plasma total homocysteine (tHcy) before and after one month treatment with 100 mg/kg/day) (A, D) or 250 mg/kg/day (B, E) in pnrCBS patients (A, B) and cblC patients (B, E) and the corresponding difference (ΔtHcy) for each pnrCBS (C) and cblC (F) patient. **Figure S3**: SAM (A, B), SAH (C, D) and the SAM/SAH ratio (E, F) after 1 month of treatment at 100 mg/kg (light grey) or 250 mg/kg (dark grey) of betaine in CBS (on left) and cblC patients (on right).

## Data Availability

Only aggregated data are being published in this study to comply with the GDPR rules.
