# Supplementary material for: Efficacy and pharmacokinetics of betaine in CBS and cblC deficiencies: a cross-over randomized controlled trial
Source: Orphanet J Rare Dis. 2022 Nov 14;17:417. doi: 10.1186/s13023-022-02567-4 (PMC9664596; doi:10.1186/s13023-022-02567-4)
Supplement: Supplementary file 1 — Additional file 1: Table S1: Patients’ characteristics. Table S2: Detail of patient current treatment. Table S3: Clinical data at inclusion. Figure S1: Carry-over effect. Figure S2: plasma total homocysteine (tHcy) before and after one month treatment with 100 mg/kg/day) (A, D) or 250 mg/kg/day (B, E) in pnrCBS patients (A, B) and cblC patients (B, E) and the corresponding difference (ΔtHcy) for each pnrCBS (C) and cblC (F) patient. Figure S3: SAM (A, B), SAH (C, D) and the SAM/SAH ratio (E, F) after 1 month of treatment at 100 mg/kg (light grey) or 250 mg/kg (dark grey) of betaine in CBS (on left) and cblC patients (on right). [file 13023_2022_2567_MOESM1_ESM.docx]

**Supplemental data**

**Supplemental data 1: detailed LC-MS/MS methods for SAM, SAH, total homocysteine, methionine, betaine, dimethylglycine and sarcosine measurement**

**SAM and SAH** were measured using a new LC-MS/MS method developed in our laboratory. Briefly, plasma, quality controls and standard were deproteinized using 10:1 30% sulfosalicylique acid. After centrifugation, 10 µL of 1 µM internal standard solution (SAH-d4 and SAM-d3) were added to 50 µL of supernatant and 500 µL of acetonitrile. The mixture was evaporated under N2 to dryness and remaining dry residues were solubilized in 250 µL of water. Ten µL were then directly injected onto Acquity UPLC I Class (Waters, Milford, USA) equipped with Acquity UPLC HSS T3 column (1.8 µm, 2.1 x 150 mm, Waters, Milford, Massachusetts). Flow rate was 0.45 mL/min with a gradient ranging from Heptafluorobutyric acid 0.1% Acetonitril:formic acid 0.1% 90:10 to 0:100 in 2.5 minutes. A Xevo-TQS (Waters, Milford, USA) triple quadrupole tandem mass spectrometer detector was operated in the positive-ion mode. Compounds were measured using the multiple-reaction-monitoring mode using the following transitions: SAH 384.95>136.15; SAM : 398.95>135.8; SAH-d4:388.95>137.97; SAM-d3:401.95>136.09). Masslynx software (Waters, Milford, USA) was used for data acquisition and processing.

**Total homocysteine (tHcy), methionine, betaine, dimethylglycine and sarcosine** were measured using a LC-MS/MS developed in our laboratory. Briefly, AF (100 µL) was mixed with 10 µL of an internal standard solution (250 µM d_8_-homocystine, 500 µM d_3_-methionine, and 50 µM d_3_-sarcosine). Bound homocysteine was released by incubating 30 minutes at room temperature with 10 µL of 500 mM DTT. After deproteinization by 300 µL of methanol samples were evaporated to dryness under nitrogen at room temperature. The dry residues were subsequently derivatized using 200 µL of 3 mol/L HCl in *n*-butanol for 15 min at 60 °C before evaporation under nitrogen at 60°C. Dry residues were solubilised in 100 µL of phosphate buffer (20 mM). Ten µL was then directly injected onto an Acquity UPLC I Class (Waters, Milford, USA) equipped with a HSS T3 column (1.8 µm, 2.1 x 100 mm, Waters, Milford, Massachusetts). Flow rate was 0.2 mL/min with a gradient ranging from Acetonitril:formic acid 0.1% 4:96 to 21.5:78.5 in 7 minutes. A Xevo-TQD (Waters, Milford, USA) triple quadrupole tandem mass spectrometer detector was used in the positive-ion mode. Compounds were measured using the multiple-reaction-monitoring mode. Masslynx software (Waters, Milford, USA) was used for data acquisition and processing.

**Supplemental table 1 : Patients’ characteristics**

|  | Disease (CblC/CBS) | Genetic charactristics Mut1/Mut2 | tHcy at diagnosis (µmol/L) | Met at diagnosis (µmol/L) | Mean tHcy in the 1 year before protocol  (µmol/L) |
| --- | --- | --- | --- | --- | --- |
| Patient 1 | CBS deficiency | ND | 350 | 770 | 49 |
| Patient 2 | CBS deficiency | ND | 380 | 450 | 84 |
| Patient 3 | cblC deficiency | p.Arg161X/ p.Arg161X | 211 | 3 | ND |
| Patient 4 | cblC deficiency | p.Trp140X/ p.Trp140X | 134 | 8 | 107 |
| Patient 5 | cblC deficiency | p.Arg91LysfsX14/ p.Trp140X | 267 | 3 | 113 |
| Patient 6 | cblC deficiency | p.Arg91LysfsX14/ p.Arg91LysfsX14 | 197 | 5.3 | 147 |
| Patient 7 | cblC deficiency | p.Arg91LysfsX14/ p.Arg91LysfsX14 | 139 | 6 | 69 |
| Patient 8 | CBS deficiency | ND | 272 | 658 | 75 |
| Patient 9 | CBS deficiency | p.Ile80Phe /p.Gly347Ser | 193 | 200 | 63 |
| Patient 10 | CBS deficiency | p.Arg121Leu/p.Ile278Thr | 268 | 94 | ND |
| Patient 11 | cblC deficiency | p.Arg91LysfsX14/ p.Arg91LysfsX14 | 170 | 8 | 99 |

**Supplemental table 2 : Detail of patient current treatment**

|  | Disease (CblC/CBS) | Current age | Diet | | Betaine  mg/kg/day | B6  mg/day | B12 vitamin supplementation | Folinic acid mg/day | Folic acid mg/day | Cysteine mg/day | Methionine mg/day |
| --- | --- | --- | --- | --- | --- | --- | --- | --- | --- | --- | --- |
|  |  |  | Protein restriction (g/day) | Amino acid mixture  (g/day of protein equivalent) |  |  |  |  |  |  |  |
| Patient 1 | CBS deficiency | 14.4 | 15.5 | 44 | 76.3 | 200 | 1 mg/d PO  Cyanocobalamine | 0.7 |  | 2000 |  |
| Patient 2 | CBS deficiency | 9.6 | 11 | 11 | 107.5 | 200 | 1 mg/d PO  Cyanocobalamine | 0.7 |  | 2000 |  |
| Patient 3 | cblC deficiency | 3.6 |  |  | 137.9 | 14.3 | 3mg/w IM  Hydroxocobalamine | 0.7 |  |  | 50 |
| Patient 4 | cblC deficiency | 4.2 |  |  | 88.9 |  | 1mg/w IM  Hydroxocobalamine | 35 |  |  | 20 |
| Patient 5 | cblC deficiency | 9.4 |  |  | 190.7 | 125 | 1mg/w IM  Hydroxocobalamine | 35 |  |  | 200 |
| Patient 6 | cblC deficiency | 17.8 |  |  | 150.9 |  | 1mg/w IM  Hydroxocobalamine | 35 |  |  | 150 |
| Patient 7 | cblC deficiency | 6.5 | 25 |  | 272.1 | 100 | 2mg/w IM  Hydroxocobalamine | 35 |  |  |  |
| Patient 8 | CBS deficiency | 13.4 | 14 | 50 | 92.6 | 250 | 1mg/d PO  Cyanocobalamine |  | 35 | 3000 |  |
| Patient 9 | CBS deficiency | 6.8 | 12.5 | 30 | 160.0 | 250 | 1mg/d PO  Hydroxocobalamine | 0.7 |  | 2000 |  |
| Patient 10 | CBS deficiency | 13.3 | 17 | 15 | 80.5 | . | 0.4 mg/d PO  Hydroxocobalamine |  | 35 |  |  |
| Patient 11 | cblC deficiency | 1.5 |  |  | 94.3 |  | 1mg/d PO  + 2mg/w IM  Hydroxocobalamine |  | 35 |  |  |

**Supplemental table 3 : Clinical data at inclusion**

|  | CBS déficiency  N= 5 | cblC deficiency  N= 6 | All patients  N=11 |
| --- | --- | --- | --- |
| Antecedent of vascular event | 3 | 0 | 1 |
| Neurologic examination   - History of vascular cerebral event - Epilepsie - Psychomotor retardation | 2  1  3 | 0  1  4 | 2  2  7 |
| Ophthalmic examination   - Retinopathy - Ectopia lentis - nystagmus | 0  5  0 | 5  0  6 | 5  5  6 |
| Bone/skeletal examination   - Cyphoscoliosis | 1 | 0 | 1 |

**Supplemental Figure 1 : Carry-over effect**

Carryover effects were tested on plasma total homocysteine, measured at each period’s baseline, between the two sequences AB/BA, where AB = 100mg/kg/day + 250mg/kg/day and BA=250mg/kg/day + 100mg/kg/day.

A p-value of 0.28, in the preliminary test for carryover, showed a non-significant difference between the two sequences. Hence, the effect of the treatment given in the first period didn’t persist into the second period and so it didn’t distort the effect of the second treatment.

Thus, the data from both periods were combined and analysed to estimate the effect of the treatment in plasma total homocysteine.

**Supplemental figure 1** shows the box plots of the plasmatic total homocysteine between the sequences AB ( light grey) and BA (dark grey) at each period, measured at the corresponding period’s baseline. Points above and below the whiskers indicate outliers outside the minimum and maximum values. Numbers of included participants per sequence at each period are shown at the bottom within the graph. Solid lines connect the mean values of plasma total homocysteine between periods for each sequence.


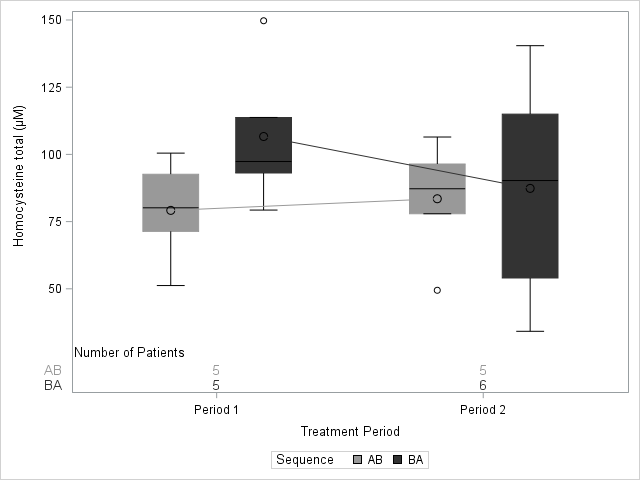


**Supplemental Figure 2 :** plasma total homocysteine (tHcy) before and after one month treatment with 100 mg/kg/day) (**A;D**) or 250 mg/kg/day (**B;E**) in pnrCBS patients (**A;B**) and cblC patients (**B;E**) and the corresponding difference (ΔtHcy) for each pnrCBS (**C**) and cblC (**F**) patient.

**100 mg/kg/day**

**250 mg/kg/day**

** Δ tHcy (µM)**

**100 mg/kg/day**

**250 mg/kg/day**

** Δ tHcy (µM)**


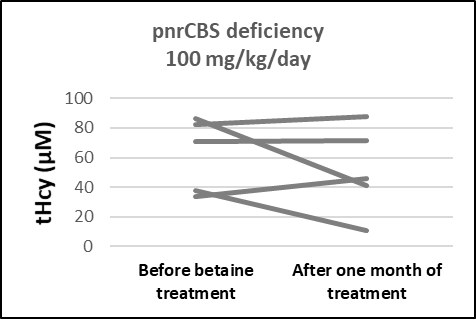

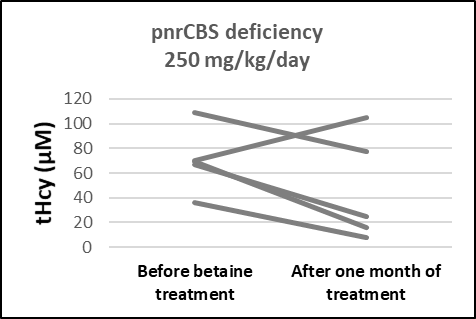

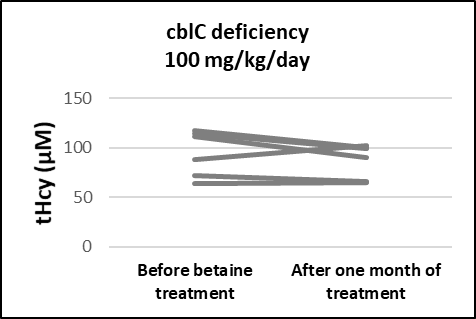

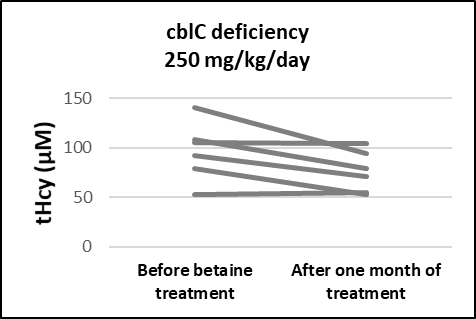


A.

B.

C.

D.

E.

F.

**Supplemental Figure 3 :** SAM (figure A & B), SAH (figures C & D) and the SAM/SAH ratio (figures E & F) after 1 month of treatment at 100mg/kg (light grey) or 250mg/kg (dark grey) of betaine in CBS (on left) and cblC patients (on right).

**A**

**B**

**D**

**C**

**F**

**E**
